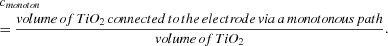# Nanoscale Analysis of a Hierarchical Hybrid Solar Cell in 3D

**DOI:** 10.1002/adfm.201302836

**Published:** 2014-02-12

**Authors:** Giorgio Divitini, Ole Stenzel, Ali Ghadirzadeh, Simone Guarnera, Valeria Russo, Carlo S Casari, Andrea Li Bassi, Annamaria Petrozza, Fabio Di Fonzo, Volker Schmidt, Caterina Ducati

**Affiliations:** Department of Materials Science & Metallurgy, University of Cambridge27 Charles Babbage Road, CB3 0FS, Cambridge, UK; Institute of Stochastics, Ulm UniversityHelmholtzstrasse 18, 89069, Ulm, Germany; CNST – Center for Nano Science and Technology @PoliMi, Istituto Italiano di Tecnologiavia Pascoli 70/3, I-20133, Milano, Italy; Department of Energy and NEMAS – Center for NanoEngineered Materials and Surfaces, Politecnico di Milanovia Ponzio 34/3, I-20133, Milano, Italy

## Abstract

A quantitative method for the characterization of nanoscale 3D morphology is applied to the investigation of a hybrid solar cell based on a novel hierarchical nanostructured photoanode. A cross section of the solar cell device is prepared by focused ion beam milling in a micropillar geometry, which allows a detailed 3D reconstruction of the titania photoanode by electron tomography. It is found that the hierarchical titania nanostructure facilitates polymer infiltration, thus favoring intermixing of the two semiconducting phases, essential for charge separation. The 3D nanoparticle network is analyzed with tools from stochastic geometry to extract information related to the charge transport in the hierarchical solar cell. In particular, the experimental dataset allows direct visualization of the percolation pathways that contribute to the photocurrent.

## 1. Introduction

Hybrid solar cells, e.g., photovoltaic devices based on heterojunctions between polymers and metal oxide nanostructures, have been proposed as viable technologies for large scale, inexpensive production of electricity from sunlight.[1–3] Even though much has been done to improve their performance and commercial viability, their efficiency is still considered too low to compete with established silicon-based technologies. Due to their composite nature and to the interplay of different components in generating a photovoltaic response, many issues need to be identified, quantified, and resolved to bring these devices to fruition.

The morphology of the nanostructured photoanodes used in hybrid solar cells is one of the main properties influencing their behavior, by affecting processes such as light scattering and charge generation, injection and transport.[1,4,5] Recent studies[6,7] have demonstrated that tailoring the interaction between the polymer and metal oxide is critical to improving the performance of hybrid solar cells. However, this tailoring can only proceed from a quantitative understanding of the behaviour of the two components at the nanometer lengthscale.

Polymer infiltration into the nanostructured inorganic component is generally difficult to achieve because of the small pore size and complex, random 3D nanostructure. Solutions to this problem include the co-processing of organic and inorganic precursors to obtain interconnected networks, demonstrated for semiconducting sulfides.[8,9] Here we investigate the use of hierarchically-structured TiO_2_ films as photoanodes, where the nano- and meso-scale porosity is obtained by tuning macroscopic growth parameters in pulsed laser deposition.[10] The novel photoanode architecture has shown promising results in dye sensitized solar cells (DSSC) achieving good infiltration of the liquid electrolyte, as well as efficient electron transport.[11]

Conventional studies of hybrid solar cells investigate optical and electrical properties, either through bulk measurements or through microtips that allow analysis of selected areas.[12] This approach however only provides indirect information on structural and physico-chemical properties of the cell, and is generally not spatially resolved at the nanometer lengthscale.

Electron tomography can be used to produce unambiguous 3D representations of nanostructures materials, where individual particles and interfaces can be distinguished and analyzed with statistical tools.[13] Successful examples of the application of electron tomography[14,15] to the analysis of hybrid solar cells include the study of ZnO-polymer,[16,17] all-polymer[18,19] and CdSe-based[20] devices, but these examples are limited to the analysis of active layer thicknesses of about 200 nm, due to the requirement for the specimen to be electron transparent at high tilt angles.

In order to produce electron transparent cross-sectional specimens of hybrid solar cells, focused ion beam (FIB) milling is generally used to create lamellar sections of the device that are thus transferred to a TEM support to be analyzed in transmission.[21,22] This sample geometry, however, is not optimal for electron tomography, since the specimen's projected thickness increases rapidly with increasing tilt, adversely affecting the quality of the dataset.[22–24] This results in deformation of the reconstruction and in the appearance of specimen projection artifacts which hinder quantitative analysis. Quantitative electron tomography was demonstrated by Biermans et al. on a micropillar-shaped cross section of a nanoporous specimen.[24]

It is important to note that this specimen geometry is ideal for electron tomography because the cylindrical symmetry and constant section of the specimen maximize the available tilt range (in principle up to 360 degrees with purpose-built specimen holders) and considerably improve the quality and reproducibility of the 3D reconstruction over established lamella-based tomography.

Our analysis of the hierarchical device uses 2D and 3D nanoscale characterization tools to evaluate the physical properties of the device. In particular we prepare a cross section of the hierarchical solar cell in the micropillar geometry, and produce a 3D reconstruction of the device using electron tomo­graphy. We employ statistical analysis and tools from stochastic geometry[25] to extract information on how photo-generated electrons can percolate through the semiconducting, nanoparticle-assembled layer. In particular, we consider spherical contact distances[16] to the TiO_2_ phase (which are related to exciton quenching efficiency) as well as geometric tortuosity[26] of the TiO_2_ phase which describes the windedness or the detours of the percolation pathways, respectively. The results indicate that the tree-like structure of the photoanode is beneficial for electron transport.

## 2. Solar Cell Structure

In the present work, we analyze a solar cell photoanode based on a hierarchical nanoparticle-assembled TiO_2_ film. The hierarchical photoanode, labeled here as h-TiO_2_, exhibits a forest-like architecture obtained by pulsed laser deposition (PLD).[10] In this technique, a TiO_2_ target is ablated by ns laser pulses, producing a plasma that expands in an oxygen atmosphere. Upon collision among the ablated species and the background gas, nano-aggregates form in the gas phase. In a pressure range from about 5 to 40 Pa, particles of around 10–20 nm in size self-assemble on the substrate in hierarchical quasi-1D structures resembling trees.[10] A post-deposition annealing treatment in air at 500 °C for 2 h is employed to obtain nanoparticles with crystalline anatase phase. The layer density, degree of branching, as well as the crystallographic phase of the individual particles, can be tuned by adjusting the deposition parameters, such as the background pressure in the deposition chamber.[10] The resulting n-type semiconducting nanostructured film features a hierarchical structure, which combines a high surface area (around 40–80 m^2^/g depending on deposition pressure[11] with a structured 3D backbone to enable charge transport.

**Figure 1:**
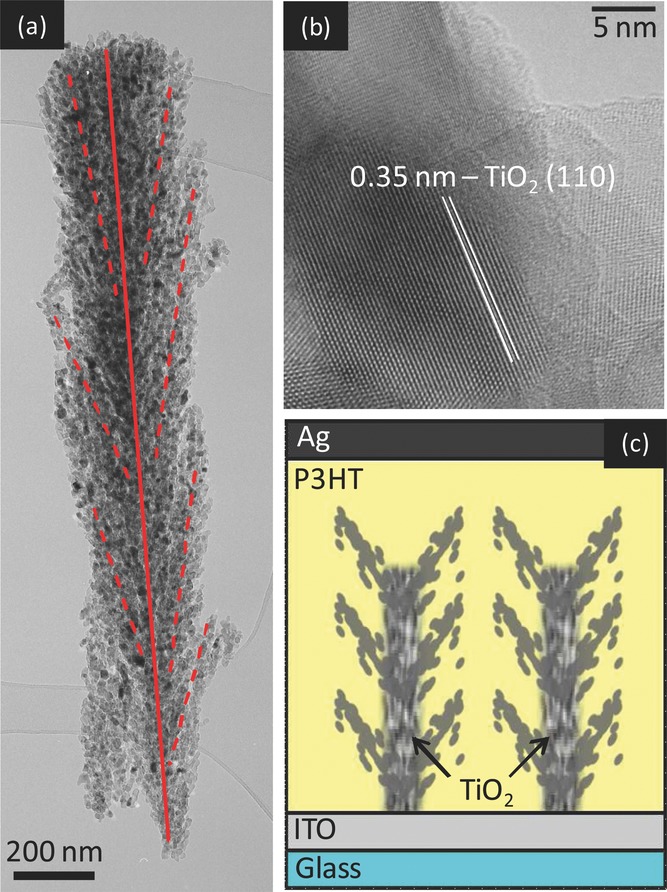
Structure of the h-TiO_2_ photoanode. a) TEM image of an individual hierarchical titania “tree” with the main body and the side branches highlighted in red. b) High-resolution TEM image of the TiO_2_ grains, showing highly crystalline anatase particles. c) Simplified model structure of the photoanode: the titania trees are separated by channels which the polymer can infiltrate.

The scanning electron microscope (SEM) images in **Table 1** show the structure of the photoanodes: a glass slide coated with a transparent conductive oxide is used as the substrate for the nanostructured h-TiO_2_. High resolution transmission electron microscopy (TEM, **Figure 1**a,b) was used to investigate grain size and crystal structure of a photoanode grown in a 20 Pa oxygen atmosphere. Individual anatase TiO_2_ grains, 12.8 ± 4.0 nm in diameter, are highly crystalline, with irregular but clean surfaces. Compared to conventional networks of randomly distributed particles, this arrangement contains vertical empty channels (Figure 1c) that are expected to favor the infiltration of the film with a p-type photo-active polymer,[27] while the 3D vertical hierarchical structure gives a well defined, branched structure of preferential conductive paths. The mechanical robustness of the individual tree-like structures, which can be removed from the substrate for TEM imaging without apparent damage, suggests good cohesion between individual grains, even though the interface between adjacent grains does not show a consistent preferential ordering.

**Table 1:** Structure and performance of a hierarchical photoanode compared to a reference paste

Cross sectional SEM Images	J_SC_ [mA/cm^2^]	V_OC_ [V]	FF	R_shunt_ [KΩcm^2^]	R_series_ [KΩcm^2^]	η [%]
	PLD h-TiO_2_					
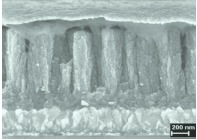	2.28	0.55	49	1.6	60	0.66
	TiO_2_ paste					
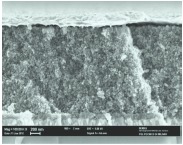	1.55	0.49	54	2	101	0.43

Thanks to these features, this structure has demonstrated potential advantages when employed in liquid dye-sensitized solar cells, showing an electron lifetime more than 1 order of magnitude longer in comparison to standard, randomly assembled TiO_2_ nanoparticle pastes, and a better interdiffusion of ionic liquid electrolytes due to its mesoporous structure.[10]

Due to the interplay between nanoscale morphology and potential benefits to the photovoltaic performance, we set out to investigate the h-TiO_2_ behaviour in solid-state hybrid cells. The prototype cell presented here comprises an ITO-coated glass substrate, a 1 μm thick h-TiO_2_ film, a spin coated P3HT layer (molecular weight 80 kg/mol) and an Ag back electrode. The thickness of the active layer was chosen as the best performing in the 0.4–1.6 μm range (Supporting Information Figure S1).

Table 1 shows the photovoltaic (macroscopic) performance of the solar cell developed from the optimization of the original h-TiO_2_ prototype (using slightly different PLD parameters and device structure, see experimental section). The h-TiO_2_ device shows a 50% improvement respect to that of TiO_2_/P3HT devices based on mesoporous photoanodes made by doctor blading deposition of commercial pastes of comparable thickness. Further improvements can be expected by optimizing the deposition and packaging process, which is beyond the scope of the present study. It is worth noting that the observed improvement is mainly driven by an enhancement in the generated photocurrent (see the Experimental Section). Both the series and shunt resistance are lower for the h-TiO_2_ device, with a combined result of a lower fill factor.

Time resolved photoluminescence spectroscopy did not show any significant change in the exciton quenching dynamics, indicating that the charge generation mechanism at the interface is not affected by the new architecture, at least in a scale comparable to the exciton-diffusion length. UV-vis absorption measuraments show that, in the range 400–650 nm, the h-TiO_2_ has a slightly lower optical density than TiO_2_ paste of comparable thickness. This suggests that the improvement in photogenerated current observed for h-TiO_2_ is even more significant.

**Figure 2:**
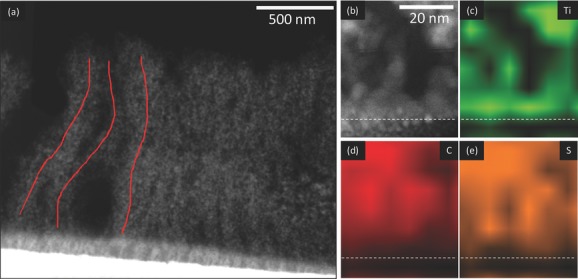
a) Cross-sectional STEM HAADF view of the photoanode, with some of the trees highlighted in red. b–e) Dark field STEM and X-ray elemental maps (respectively Ti, C and S) from the base of the TiO_2_ structures, showing presence of sulphur and indicating a good infiltration of P3HT inside the titania network. The white dashed line outlines the surface of the substrate.

We verify P3HT infiltration in the h-TiO_2_/P3HT cell by using the FIB to prepare a cross sectional lamella of the solar cell (about 150 nm thick) to investigate the polymer infiltration. **Figure 2**a,b show high angle annular dark field (HAADF) scanning transmission electron microscope (STEM) image of the cross sectional cell, with panels (c,d) containing information on the spatial distribution of Ti, C, S, respectively, as obtained by energy dispersive X-ray spectroscopy (EDS). The presence of the polymer is revealed by the sulfur signal, detectable in depth down to the ITO layer, at least in the wider channels. Due to the sensitivity of the material to the 200 kV electron beam, the elemental maps were acquired with low sampling (large pixel size), and short integration time.

## 3. Nanoscale 3D Structure

We applied HAADF STEM tomography to further investigate the 3D morphology of the solar cell. The geometry of the specimen is a crucial parameter in this technique, as it determines the effective available tilt range during acquisition, and affects the quality of the dataset. Since our aim was to obtain high quality quantifiable data with minimal operator input and bias, we adopted a micropillar geometry for the cross sectional specimen preparation, rather than the conventional lamella geometry.

**Figure 3:**
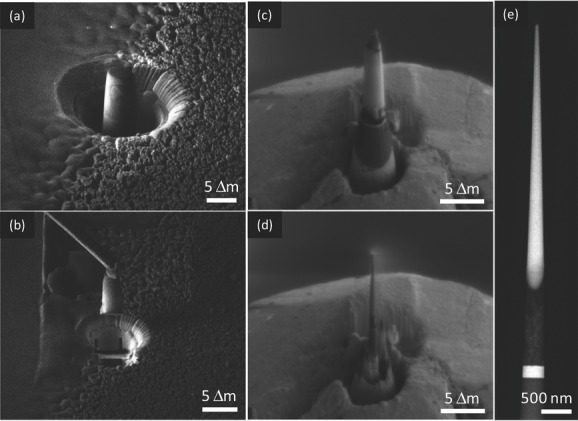
Focused ion beam preparation of the sample for electron tomography. a) A pillar is isolated from the photoanode and b) extracted with a micromanipulator. It is then positioned on a TEM-compatible mount (c) and thinned until electron transparency is achieved (d). e) The micropillar as observed in the TEM.

From the complete h-TiO_2_/P3HT device (except for the Ag electrode) we select a feature on the film and prepare a tapered micropillar sample (6 μm long and 300 nm thick at the base). **Figure 3** shows the various steps of the FIB preparation, ending with the micropillar attached to the tip of a tomography pin. To preserve the device structure while achieving electron transparency, this specimen preparation approach requires very high spatial accuracy, since the diameter of the pillar is roughly the same as that of the branched nanostructures in the titania film (i.e., about 200 to 500 nm).

**Figure 4:**
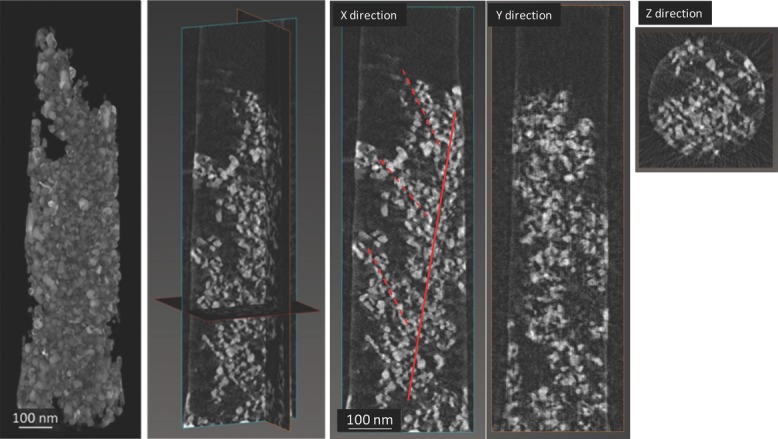
Reconstructed volume of the micropillar after electron tomography, shown in 3D rendering (left panel) and orthogonal slices. The branched meso-structure is highlighted in red in the slice taken along the X direction.

The micropillar/pin assembly is then mounted onto a tomography pin holder. We acquire images in HAADF STEM mode, collecting a signal which is sensitive to the atomic number of the specimen. Given the composite nature of the specimen and the large variation in average Z-number for different regions, care must be taken to optimize the brightness and contrast levels in the high angle annular dark field images, thus allowing for efficient discrimination of all the solar cell components. To fully determine how the hierarchical structure develops across the thickness of the device, we select a magnification value (80k×) that allows us capture the whole micropillar. A series of images is acquired at various degrees of tilt along the micropillar's longitudinal axis (see the Experimental Section). 3D reconstruction and image segmentation, usually some of the most tedious and potentially operator-biased steps of electron tomography, are relatively straightforward on our dataset thanks to the quality of the tilt series obtainable from a sample with cylindrical symmetry.[23,24] A reconstructed cross-section is reported in **Figure 4**a, and a volume rendering in Figure 4b. The minimal manual input for the volume segmentation, in particular, ensures the reliability and reproducibility of the quantitative analysis of the reconstruction.

The 3D reconstruction shows a structure in which the TiO_2_ particles aggregate to form a central column 100 to 150 nm wide surrounded by side branches pointing upwards (away from the ITO layer), and is therefore representative of the photoanode (a larger section of which is shown in Figure 2a). P3HT completely infiltrates the pores in the structure, down to the ITO–TiO_2_ interface. The TiO_2_ structure shows some damage towards the external part of the micropillar, limited to a depth of about 20 nm from the surface, due to interaction with the gallium beam during the FIB processing.

## 4. Quantitative Analysis

The TiO_2_ volume fraction is estimated as 19 ± 2% of the micropillar volume. We find a surface area value of 82 ± 4 m^2^/g, assuming that the titania is entirely in the anatase phase (3.86 g/cm^3^). This value is in good agreement with BET measurements on PLD TiO_2_ hierarchical films before polymer infiltration.[11] Since BET measurements cannot be performed on films once they have been infiltrated with a polymer, electron tomography is vital in confirming that the roughness of the surface is preserved throughout the device assembly process, including the polymer infiltration. The size distribution of the titania particles can also be extracted (shown in the Supporting Information), finding an average particle size of 12.3 ± 3.8 nm, in good agreement with the high resolution TEM measurements carried out on the pristine TiO_2_ film, which indicated a size of 12.8 ± 4.0 nm (see Supporting Information Figure S2).

Subsequently we apply descriptive statistical analysis[16,25] to analyze the 3D morphology. First of all, we measure the connectivity of the TiO_2_ particles, i.e., the fraction of TiO_2_ connected with the electrode.[16] In this kind of solar cell, the electron–hole pairs are photo-generated in the polymer layer, and they separate at the TiO_2_–P3HT interface. The electrons are injected into the titania structure and have to travel to the transparent electrode, shown at the bottom in our images. Therefore, the efficiency depends on the existence of unhindered percolation pathways to transport charges towards the electrodes. It is found that 98.5% of the TiO_2_ is connected with both electrodes (located at the top and bottom). Moreover, these pathways should preferably be monotonic; this measure of the connectivity is therefore direction-dependent, particularly in a branched or dendritic structure (see Supporting Information Figure S3). For electron transport, the monotonic connectivity of h-TiO_2_, defined as the fraction of TiO_2_ material connected to the anode through a path with strictly decreasing distance, is found to be 89.5% (see Supporting Information Figure S3) for the volume considered in this calculation. When the conductive paths are evaluated in the opposite direction, only 69.6% of the material is monotonically connected (towards the top). Such a difference suggests that the hierarchical titania structure favors electron transport towards the anode, thus minimizing losses due to recombination. The h-TiO_2_ film can be considered as the multibranched analogue of TiO_2_ rods. Hence, a type of asymmetrical, channeled conduction is expected, different from the symmetrical percolative conduction typical of photoanodes based on randomly assembled nanoparticles.

**Figure 5:**
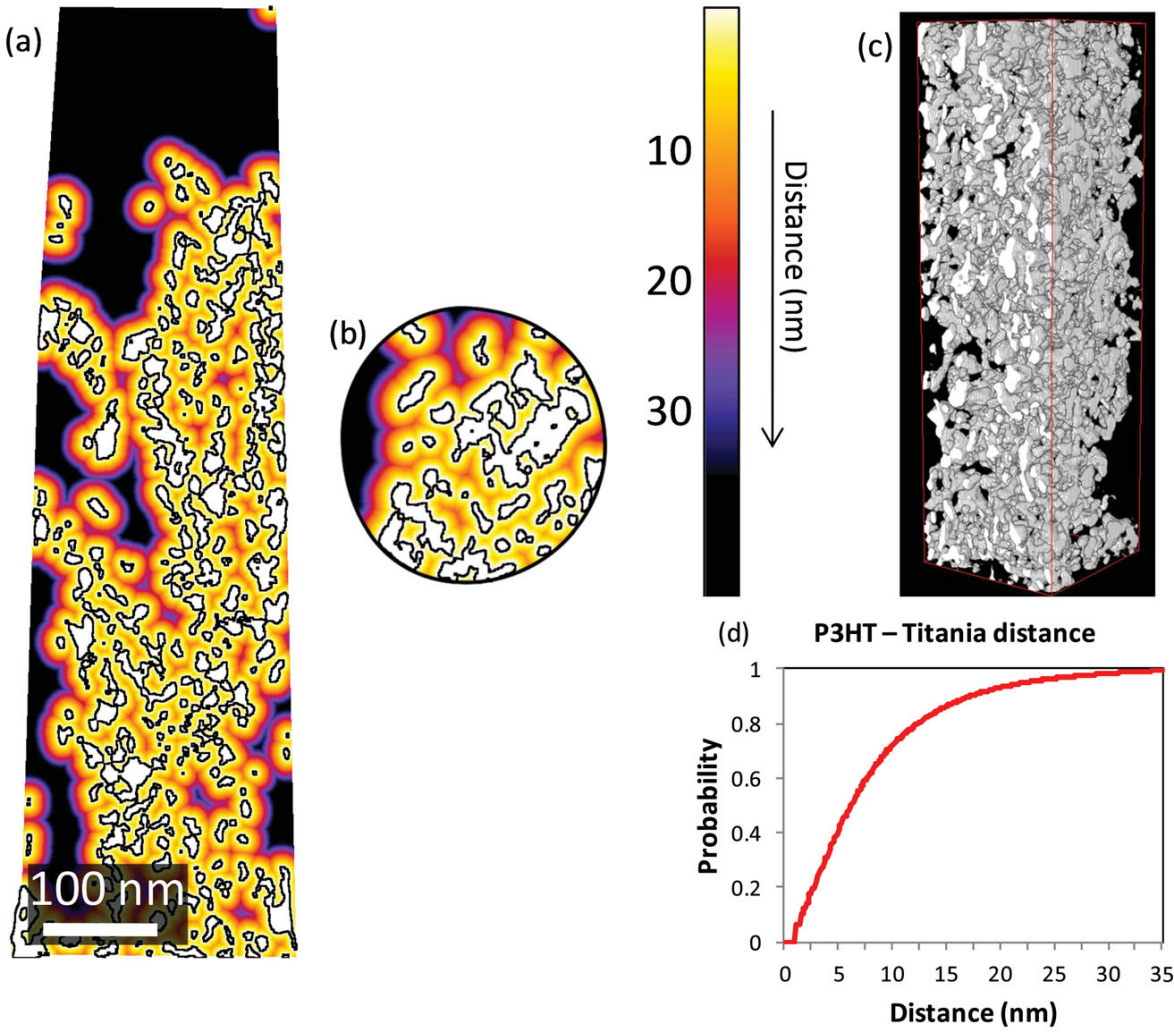
Map of the distance from the P3HT voxels to the titania in cross-section taken a) from the side and b) from the top. c) Subvolume considered for the distribution of the spherical distances between P3HT voxels and the nearest titania particle. d) Corresponding spherical contact distribution function *D*:[0,∞]→[0,1], where *D(r)* denotes the probability to reach the TiO_2_ phase from a random location in the pore phase. The exciton diffusion length in P3HT is roughly up to 8.5 nm.

The properties of the pore space between the TiO_2_ particles can be analyzed to determine the possibility for charge injection into the semiconducting layer. In particular, in the 3D reconstruction it is possible to measure the distance between any point in the pores and the nearest TiO_2_ particle. In case of a perfect infiltration, in which P3HT entirely fills the pores in the TiO_2_ film, this corresponds to determining how much polymer is close enough to the P3HT/TiO_2_ interface to inject a photo-generated electron. To optimize the performance of the solar cell, this distance has to be smaller than, or at least comparable with, the exciton diffusion length in P3HT (values between 4 and 8.5 nm are found in literature.[28] We select a sub-volume (**Figure 5**c) of the TiO_2_ pillar to investigate this structural property. For our system, by considering spherical contact distances,[25] it is found that 75.8% of the pore space voxels are within 8.5 nm from the interface (Figure 5). A color-coded map of the distance between pore voxels and the TiO_2_ surface is reported in Figure 5, showing how the h-TiO_2_ morphology can interact with the polymer phase more effectively. This visualization is helpful in identifying the areas in the film where the intermixing between the two phases is not sufficient to generate free charges. We note that this type of information can only be retrieved from a 3D dataset with nanometer spatial resolution, such as electron tomography.

**Figure 6:**
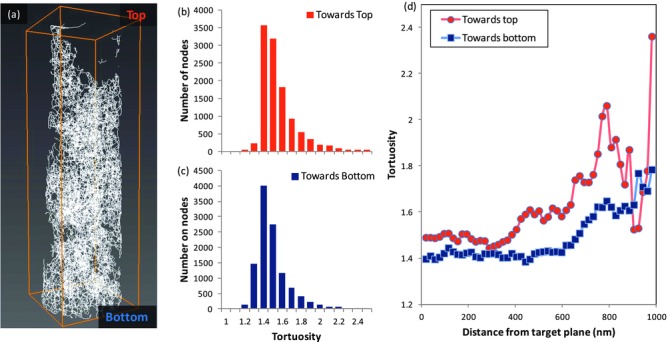
Geometrical properties of the titania network (a). In (b) and (c) the distribution of geometric tortuosities for the two directions are reported, showing the anisotropy of the system. d) Evolution of the tortuosity through the network for both directions.

In the following, we analyze the geometry of the electrons pathways along the TiO_2_ network towards the electrode, in particular their complexity. For different positions where electrons can be generated in the network, we compare the shortest distance the electron has to travel along the network of TiO_2_ particles to reach the electrode with the distance in a straight (vertical) line. This yields the geometric tortuosity,[26] defined as the ratio between the length of the shortest path divided by the orthogonal distance along the z-axis. Through a skeletonization algorithm, we reduce the particle network to a graph representation consisting of nodes and edges (**Figure 6**), for which we compute the geometric tortuosity,[26] where we consider shortest paths starting from nodes of the graph located anywhere in the material to the electron collecting electrode.

Geometric tortuosity is equal to 1 for systems in which there are ideal vertical paths for charge collection (for example in the case of vertically aligned nanowires, or a bulk non-porous film); higher values reflect a more complex geometry, where flow occurs through convoluted paths.

This characteristic parameter provides another indication of the behavior of h-TiO_2_ in terms of electronic transport. We find values that are close to those observed in micro-porous systems with comparable porosity (around 1.0–2.0);[26,29] however, unlike what happens in isotropic micro- and nanostructures, the tortuosity is not symmetrical in our network. From the whole skeleton, we find 1.466 ± 0.003 for conduction going towards the anode and 1.559 ± 0.003 in the opposite direction, suggesting that the hierarchical ordering helps optimizing the electron transport. The uncertainties reported are the standard errors of the mean for the measurement of the average, which is done on about 11 000 nodes.

## 5. Conclusions

We characterized a promising model hybrid solar cell system, based on a P3HT-infiltrated TiO_2_ photoanode produced via pulsed laser deposition, featuring large surface area and a hierarchical structure, properties of paramount importance for solar cell photo­anodes. Polymer infiltration is verified at the nanoscale. We demonstrate a quantitative assessment of the polymer infiltration in the hierarchical TiO_2_ film, showing how electron tomography performed on a FIB-prepared micropillar specimen provides complete morphological information on a nanostructured composite device, with minimal user input. We also demonstrate how statistical analysis of the electron tomography dataset using tools from stochastic geometry provides quantitative information on transport pathways towards the anode, and on the geometry of the interface between the organic and inorganic phases in the hybrid solar cell.

The methodology for specimen preparation, characterization, and analysis, can be applied to other systems, particularly in the field of photovoltaics and catalysis. We expect the combination of FIB micropillar sample preparation, electron tomography and statistical analysis to become a standard tool to study solar cell model systems and contribute to the design of novel groundbreaking devices.

## 6. Experimental Section

*Synthesis via PLD and Device Assembly*: Pulsed laser deposition (PLD) of titanium oxide was accomplished by ablating a 99.99% pure TiO_2_ target with laser pulses from a Nd:Yag in 4^th^ armonic (wavelength 266 nm, duration 7 ns, energy density 4 J cm^−2^, 10 Hz repetition rate) in 7 Pa O_2_ background pressure. PLD in a background atmosphere can lead to cluster nucleation in the ablation plasma plume and soft landing of clusters on the substrate, depending on deposition parameters, as extensively described in previous articles.[10,30–32]

TiO_2_ films were grown on ITO-glass substrates at room temperature, yielding amorphous films. Post-deposition annealing in air in a muffle oven at 500 °C for 2 h was performed in order to obtain crystalline films, mainly composed of anatase. Crystallinity was characterized (not shown) by X-ray diffraction (Philips PW3020, Cu Kα radiation) and Raman spectroscopy (Renishaw InVia, excitation wavelength 514.5 nm).

*Sample Preparation*: We used a FEI Helios Nanolab dual beam machine, with a Ga^+^ ion beam for selectively milling areas of the specimen and a FEG emitter for electron imaging during the milling process. In this way we first surveyed the solar cell structure and isolated the representative features on the film. We first deposited a protective layer of platinum on the area of interest and applied a rough cut using annular milling with a current of 2.8 nA at 30 kV, isolating a rod of about 5 μm in diameter. It is important in this process to mill through to the glass substrate, so that the entire device section is extracted. We then used the Omniprobe micromanipulator to move the rod from the device to the apex of a TEM-compatible tungsten pin. Ion-beam Pt deposition was used to secure the rod to the W pin, and then the Ga ion beam was used to thin the rod by annular milling, progressively decreasing the beam current to reduce ion-beam damage, ending at 9 pA. Finally, a micropillar of about 300 nm in diameter was obtained (for the materials in the solar cell this corresponds to a good compromise between sampling volume and electron transparency). This milling procedure ensures that the long axis of the pillar is parallel to the main axis of the W pin, and it is essential to minimize misalignments in the tomographic acquisition.

*Electron Microscopy*: A tilt series was acquired in a FEI Tecnai F20 (200 kV acceleration voltage) using a Fischione on-axis pin holder. The tilt range is from –72 to +72 degrees, with an image acquired every 2 degrees. A tilt range of 140 degrees or more has been shown to limit “missing wedge” artifacts, resulting in a good spatial resolution and low elongation of features.[23] Although with the micropillar specimen, it would –in principle- be possible to record a full tilt series, our particular pin holder did not allow sufficiently accurate positioning of the pin over the full tilt range. Given that inaccuracies in angular positions can adversely affect the quality of the reconstruction, we did not make use of the full tilt series. Our tilt range results in an estimated elongation error of up to 10% for measurements of linear dimensions along the beam direction.[23] This causes a maximum error of 10% on volumes, and 5% for surfaces.

The imaging signal was collected using a high-angle annular dark field (HAADF) detector, providing atomic-number contrast. The magnification is 80 kx and the pixel size is 0.77 nm. The images are aligned using Inspect3D and the reconstruction (shown in Supporting Information Video S4) is computed with the same software using a weighted back projection (WBP) algorithm. It is known that, as a general rule, iterative algorithms (like SIRT) yield a more accurate reconstruction, particularly in the case of a large missing wedge. However, such algorithms require a large number of iterations (>30) to correctly resolve high spatial frequencies, and sometime present convergence problems. Therefore, for some datasets, WBP can return sharper reconstructions than SIRT. This is the case for the system under analysis here, composed by a network of particles which are very small compared to the overall volume.

SIRT also tends to smooth the signal, resulting in slightly smeared features compared to WBP. This makes segmentation more ambiguous, since the range of values that can be selected by an operator for a threshold in SIRT is quite broad. On the other hand, WBP results in sharp features, thus making segmentation less arbitrary. Supporting Figure 5 shows the difference in reconstruction for a slice taken along the pillar axis, including a histogram of the grayscale values and the resulting segmented dataset (threshold has been set at 47% of the grayscale range for both images, corresponding to the onset of the middle peak in the histogram for both reconstructions). The histograms are divided in three regions, corresponding to void, P3HT and TiO_2_ voxels respectively. The image segmented from the WBP dataset preserves most of the fine features, while the segmentation on the SIRT dataset fails at resolving fine details.

The quality of the reconstructions obtained with different algorithms was evaluated by comparison with the high resolution TEM data, finding a better match with the WBP algorithm.

We then processed the reconstructed volume with ImageJ to remove background fluctuations and optimize the contrast between the different components of the film. We finally applied a threshold in ImageJ to obtain a segmented dataset and use ResolveRT to visualize the reconstructed 3D volume.

*Statistical Data Analysis*: The data was smoothed using a Gaussian filter in Fiji with variance *σ*^2^ = 0.25, which is a very light smoothing operator and subsequently binarized by global thresholding. The skeletonization was performed using Avizo 6.3, using the default settings.

The content of TiO_2_ was estimated from a sub-volume (Figure 5c) of the segmented dataset. The mass was evaluated from the volume of the two phases (TiO_2_ and P3HT) using the respective densities. The specific surface area was calculated by dividing the surface measured with Avizo by the mass of the sample. The particle size was extracted by iterative erosion of the segmented dataset until the centers were isolated. The number of erosion steps, for spherical particles, is an estimate of the radius; the erosion procedure is able to resolve partially overlapping features.

The geometric tortuosity is calculated for each node *i* ∈{1,…, *n*} individually by 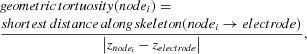
where *z* describes the z-coordinate in the 3D image. The shortest paths along the graph are determined using Dijkstra's algorithm.[33,34] Let *X* be the shortest distance from a randomly chosen point in the polymer phase to the TiO_2_ phase. Then the spherical contact distribution function *D* is given by *D*(*r*) = *P*(*X* ≤ *r*). The monotonous connectivity *c_monoton_* for the TiO_2_ phase is defined by