# Supplementary material for: Nanoscale Analysis of a Hierarchical Hybrid Solar Cell in 3D
Source: Adv Funct Mater. 2014 Feb 12;24(20):3043–50. doi: 10.1002/adfm.201302836 (PMC4376200; doi:10.1002/adfm.201302836)

Copyright WILEY-VCH Verlag GmbH & Co. KGaA, 69469 Weinheim, Germany, 2014.

ADVANCED FUNCTIONAL MATERIALS

Supporting Information

for *Adv. Funct. Mater.*, DOI: 10.1002/adfm.201302836

Nanoscale Analysis of a Hierarchical Hybrid Solar Cell in 3D

Giorgio Divitini, Ole Stenzel, Ali Ghadirzadeh, Simone Guarnera, Valeria Russo, Carlo S. Casari, Andrea Li Bassi, Annamaria Petrozza, Fabio Di Fonzo, Volker Schmidt, and Caterina Ducati*

Supplementary information 1. Photovoltaic (macroscopic) characterization of h-TiO_2/P3HT solar cells with different thickness, and comparison of the I-V curves for h-TiO_2 and paste based devices.

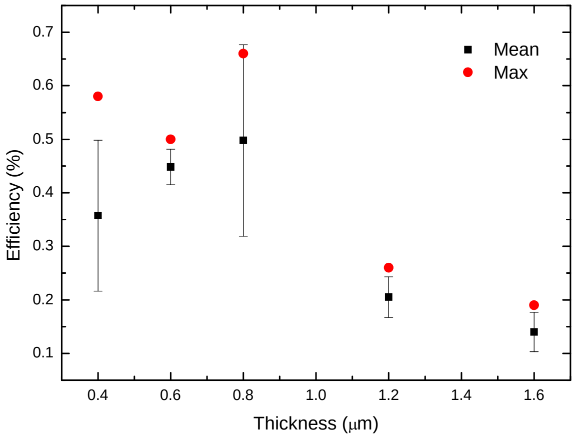

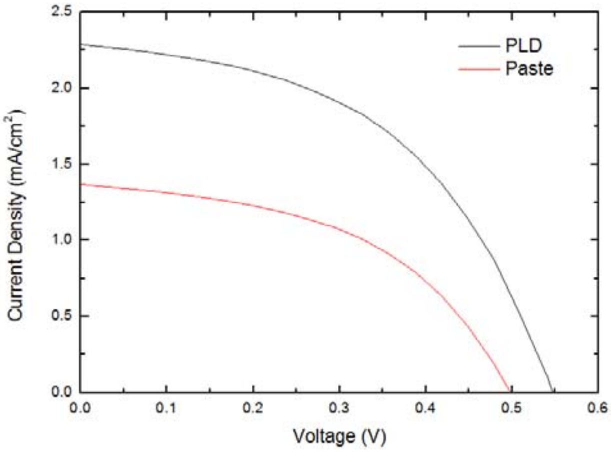

Supplementary Information 2. Size distribution of the TiO_2 particles, comparison between high resolution TEM and tomographic reconstruction (with a weighted back projection reconstruction algorithm). In electron tomography, the size has been measured by iterated erosion of the segmented dataset with ImageJ. This procedure resolves overlapping particles; the number of erosion steps required to reduce the particle to a single pixel is an estimate of the radius (in pixels), from which the size of the particle can be calculated.

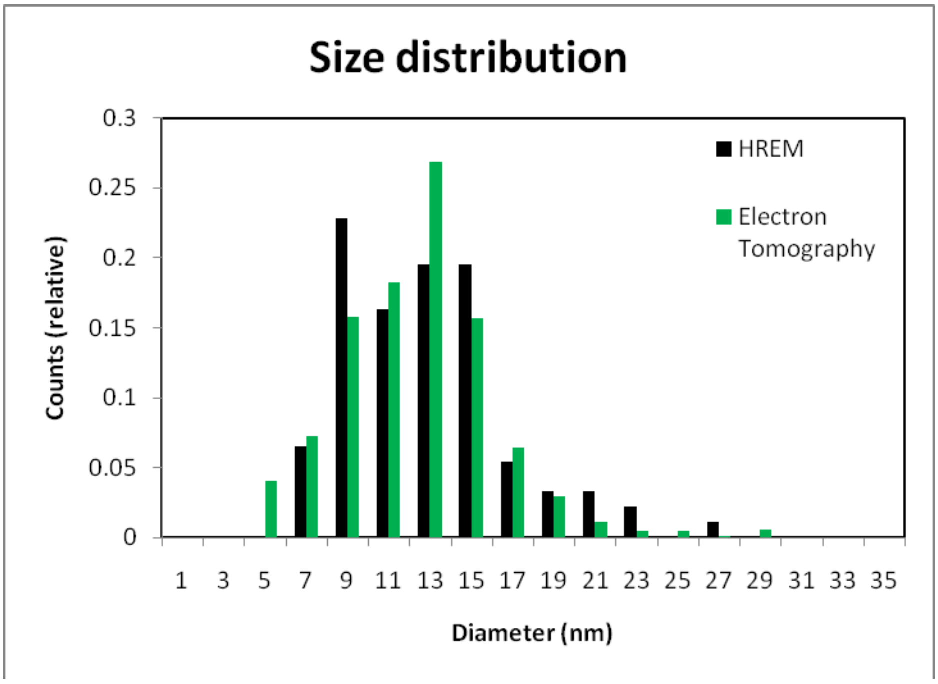

Supplementary Information 3. (Left) Sub-volume of the reconstruction used for connectivity analysis of the network. (Right) Example of evaluation of connectivity in two different directions. The grey pixels, although connected to the top/bottom planes, are not monotonically connected.

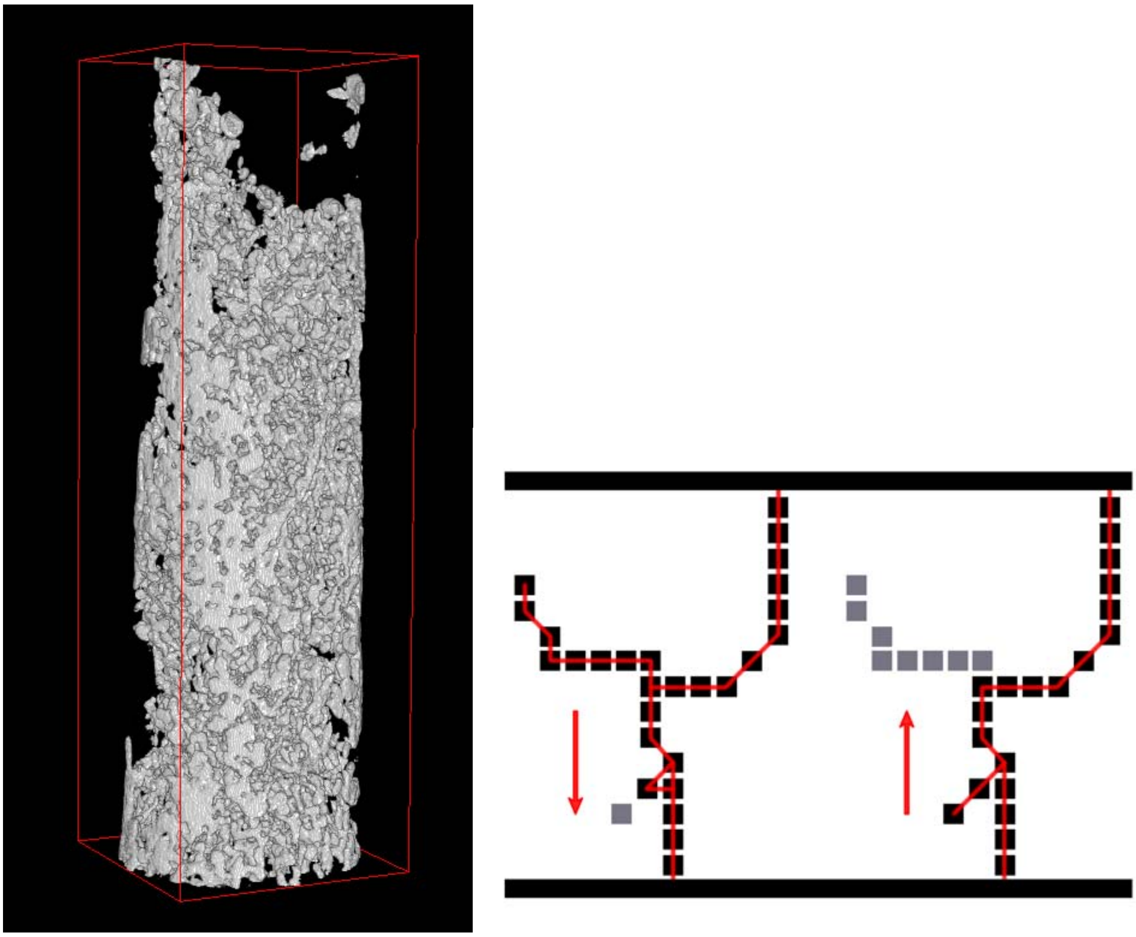

Supplementary Information 4. Animation showing the 3D rendering of the tomographic reconstruction.

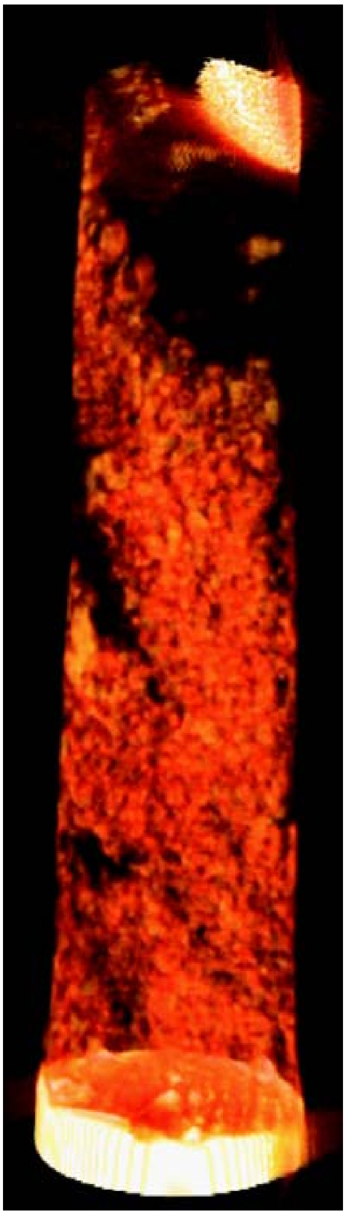

Supplementary information 5. Comparison between different electron tomography reconstruction algorithms. WBP produces sharper features, while SIRT features are smeared out, here shown in orthoslices along the *z* direction (along the tilt axis, top row, a-b). This results in a more straightforward binarization of the dataset (center row, c-d). The bottom row (e-f) shows histograms relative to the reconstructed volumes, with the vertical lines separating the voxels assigned, from left to right, to void, polymer and TiO_2 respectively.

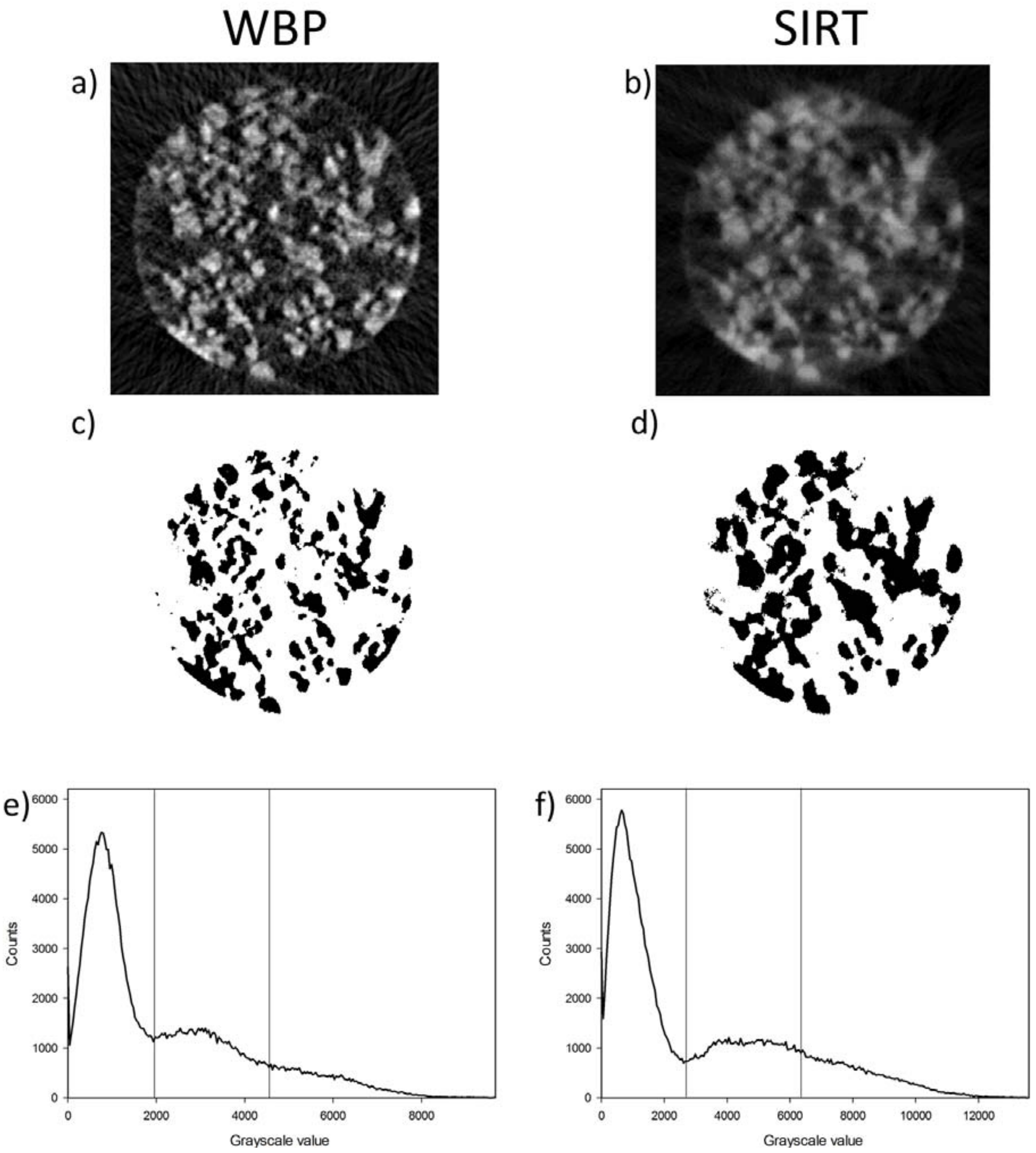

Supplement: Supplementary file 1 — Supplementary [file adfm0024-3043-SD1.pdf]
